# An in vitro mechanistic study of cholinergic-associated mechanisms relevant to MASLD in HepG2 cells

**DOI:** 10.55730/1300-0152.2806

**Published:** 2026-04-21

**Authors:** Gamze SÖNMEZ, Müslüm GÖK, Suat SARI, Ebru BODUR

**Affiliations:** 1 Department of Medical Biochemistry, Faculty of Medicine, Hacettepe University, Ankara, Turkiye; 2 Department of Biochemistry, Faculty of Medicine, Muğla Sıtkı Koçman University, Muğla, Turkiye; 3 Department of Pharmaceutical Chemistry, Faculty of Pharmacy, Hacettepe University, Ankara, Turkiye

**Keywords:** MASLD, α-linolenic acid, linoleic acid, butyrylcholinesterase, cholinergic signaling, β-oxidation

## Abstract

**Background/aim:**

Polyunsaturated fatty acids (PUFAs) and the hepatic cholinergic axis are critical regulators of lipid metabolism and inflammatory signaling; however, their time-dependent interplay in hepatocytes remains incompletely understood. Clarifying how n-6 and n-3 PUFAs interact with cholinergic pathways may provide mechanistic insight into metabolic dysfunction-associated steatotic liver disease (MASLD).

**Materials and methods:**

HepG2 cells were used to examine the combined effects of butyrylcholinesterase (BChE) overexpression and PUFA exposure. Cells were treated with linoleic acid (LA; n-6) or α-linolenic acid (α-LA; n-3) for 24 or 48 h. Gene expression analyses were performed to assess pathways involved in de novo lipogenesis, β-oxidation, cholinergic signaling, and inflammatory responses. Cholinesterase activities were measured enzymatically, secreted cytokines were profiled, and molecular docking simulations were performed to evaluate potential α-LA interactions with the α7 nicotinic acetylcholine receptor (α7 nAChR) and M3 muscarinic acetylcholine receptor (M3 mAChR).

**Results:**

An early antilipogenic profile and changes suggestive of increased fatty-acid oxidation (↓SREBP-1c, ↓ACC1, and ↑CPT1A) were induced by α-LA at 24 h and were partially attenuated by 48 h. LA exhibited a biphasic pattern characterized by modest early suppression followed by a pronounced proinflammatory profile at 48 h (↑TNF, ↑IL6, and ↑COX-2). BChE overexpression was associated with increased expression of lipogenic and triglyceride-related genes, whereas cotreatment with PUFAs resulted in time- and class-specific modulation. Cholinergic markers exhibited divergent expression patterns: BCHE + α-LA increased *CHAT* and *CHRNA7* expression at 48 h, whereas ACHE mRNA expression increased under both BCHE + LA and BCHE + α-LA conditions. Enzymatic analyses demonstrated increased total cholinesterase activity in the BChE and α-LA groups, while molecular docking supported the potential accommodation of α-LA within the binding pockets of α7 nAChR and M3 mAChR.

**Conclusion:**

PUFA class and exposure duration jointly shape hepatocellular metabolic and inflammatory states, with cholinergic signaling acting as a modulatory node relevant to MASLD.

## Graphical abstract

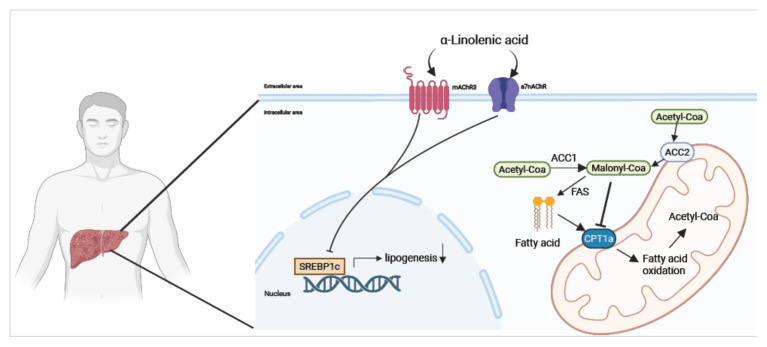

## 1. Introduction

Metabolic dysfunction–associated steatotic liver disease (MASLD) and its inflammatory subtype, metabolic dysfunction–associated steatohepatitis (MASH), are leading causes of chronic liver disease and are tightly linked to obesity, insulin resistance, and dyslipidemia (Jiang et al., 2024). Disturbances in hepatic fatty-acid handling, de novo lipogenesis, β-oxidation, and triglyceride (TG) assembly are central to disease initiation and progression, whereas inflammatory signaling contributes to the transition from simple steatosis to steatohepatitis (Steinberg et al., 2025).

Dietary polyunsaturated fatty acids (PUFAs) play critical roles in regulating these pathways. Linoleic acid (LA; n-6) and α-linolenic acid (α-LA; n-3) are essential PUFAs that serve as precursors to long-chain biologically active lipids (Simopoulos, 2002). Western dietary patterns often result in an n-6/n-3 imbalance, which has been associated with an increased risk of MASH and reduced hepatic long-chain n-3 PUFA pools in patients (Jeyapal et al., 2018). Evidence also indicates that hepatic fatty-acid composition and the expression of lipid-metabolism–related genes differ between simple steatosis and MASH, highlighting a mechanistic link between PUFA biology and disease severity (Arendt et al., 2015).

Beyond lipid metabolism, cholinergic signaling may contribute to hepatic metabolic and inflammatory control. Butyrylcholinesterase (BChE), a liver-derived enzyme included in certain liver function panels, degrades ghrelin and has been associated with insulin resistance and fatty liver traits, thereby positioning the cholinergic axis at the intersection of energy balance, appetite regulation, and hepatocellular metabolism (Chen et al., 2016). These observations raise the possibility that cholinergic tone, herein used to denote an inferred state of cholinergic activity based on cholinesterase (ChE) activity and related gene-expression patterns rather than a directly measured functional parameter, may be altered and thereby influence hepatocellular lipid metabolism and inflammatory status.

However, the crosstalk between PUFAs and the cholinergic system in hepatocytes, particularly with respect to the time dependency of these interactions, remains incompletely defined. It was previously demonstrated that treatment with 5 μM α-LA following BChE transfection in HepG2 cells increased BChE expression and activity and was accompanied by an overall increase in cell number. At this low concentration, the proportion of apoptotic cells was reduced by α-LA, whereas an equivalent concentration of LA did not produce this effect and instead induced a ghost cell–like morphology. Taken together, these findings suggest that BChE expression in HepG2 cells may be regulated by α-LA (Gok et al., 2016).

To address this gap, we used HepG2 cells as a well-established in vitro model for studying hepatic metabolism and inflammatory signaling. HepG2 cells retain key hepatocyte-like functions, including active lipid-metabolic pathways and responsiveness to metabolic and inflammatory stimuli, thereby rendering them suitable for controlled mechanistic investigations. Accordingly, it was hypothesized that PUFA class and exposure duration differentially modulate hepatocellular lipid metabolism and inflammatory responses through cholinergic pathways. Specifically, the effects of LA (n-6) and α-LA (n-3) on de novo fatty-acid synthesis, fatty-acid oxidation, TG synthesis, cholinergic markers and receptors, and inflammatory readouts were examined under early (24 h) and extended (48 h) exposure conditions. We further explored whether these metabolic and inflammatory endpoints could be altered through modulation of the cholinergic system, with potential relevance to MASLD.

## 2. Materials and methods

### 2.1. Cell culture and viability

HepG2 cells were maintained in low-glucose Dulbecco’s Modified Eagle’s Medium (DMEM) (Gibco; Thermo Fisher Scientific, Waltham, MA, USA) supplemented with 10% fetal calf serum (FCS) (Gibco; Thermo Fisher Scientific, Waltham, MA, USA), 2 mM glutamine (Lonza, Basel, Switzerland), and 1% penicillin–streptomycin (Lonza, Basel, Switzerland). Cells were subcultured upon reaching approximately 70% confluence. For viability assays, 1 × 10^4^ cells in 100 μL were seeded per well of a 96-well plate. After 24 h, the medium was replaced with fresh DMEM containing LA or α-LA at final concentrations of 0.5, 5, 50, or 500 μM. Cells were exposed to these lipids for 48 h, after which 10 μL of WST-1 reagent (cat. no. 11644807001; Roche Diagnostics, Mannheim, Germany) was added to each well. Following a 4 h incubation at 37 °C, absorbance was measured at 420 nm using a 600 nm reference wavelength with a VersaMax microplate reader (Molecular Devices, San Jose, CA, USA) to determine IC_50_ values.

### 2.2. Plasmid amplification and purification

A control plasmid, pUC19 (Clontech Laboratories, Mountain View, CA, USA), was propagated in HST08 (Stellar) competent *E. coli* cells (Clontech Laboratories, Mountain View, CA, USA), as previously described (Gok et al., 2016). For transfections, plasmid DNA was isolated by alkaline lysis using a Qiagen Midiprep kit (Qiagen, Hilden, Germany). The purified plasmids were subsequently digested with BglII (New England Biolabs, Ipswich, MA, USA) to verify the presence of the BCHE insert.

### 2.3. HepG2 cell transfection with wt-BCHE and pUC19 plasmids

Transient transfection of HepG2 cells with wt-BCHE or the pUC19 control plasmid was performed using DharmaFECT Duo Transfection Reagent (Thermo Fisher Scientific, Waltham, MA, USA). A total of 2 × 10^5^ cells suspended in 1 mL of medium were seeded into each well of a 12-well plate 24 h before transfection. After 24 h, the culture medium was removed, and transfections were performed in serum- and antibiotic-free low-glucose DMEM using 1 μg of either wt-BCHE or pUC19 plasmid DNA, according to the manufacturer’s instructions. Cells were exposed to a mixture of transfection reagent, plasmid DNA, and serum- and antibiotic-free low-glucose DMEM for 4 h, after which the medium was replaced with low-glucose DMEM supplemented with 10% FCS and 2% glutamine. For experiments involving lipid treatment, 4 h after transfection, the medium was replaced with low-glucose DMEM supplemented with 2% glutamine and 10% FCS and containing either 5 μM LA or 5 μM α-LA (Sigma-Aldrich, St. Louis, MO, USA), and the cells were cultured for up to 48 h. Cells and conditioned media were collected at predefined time points and stored at −80 °C for subsequent analyses. The 24 h and 48 h exposure periods were selected to capture early and more sustained cellular responses to treatment, respectively. BCHE overexpression was achieved through plasmid transfection to increase cellular ChE levels. The resulting increase in enzymatic activity, as measured using the Ellman assay, reflects the functional consequence of enhanced BCHE expression.

### 2.4. RNA isolation and quantitative PCR

Total RNA was extracted using TRIzol reagent (NucleoGene, İstanbul, Turkiye) according to the manufacturer’s instructions, and RNA yield and purity were assessed using a NanoDrop One spectrophotometer (Thermo Fisher Scientific, Waltham, MA, USA). For cDNA synthesis, 1 μg of total RNA per sample was reverse transcribed using a cDNA Synthesis Kit (NucleoGene, İstanbul, Turkiye). Gene expression was quantified by SYBR Green–based real-time quantitative PCR (qPCR) (Roche Diagnostics, Mannheim, Germany). Genes associated with de novo lipogenesis included acetyl-CoA carboxylase 1 (*ACC1*), acetyl-CoA carboxylase 2 (*ACC2*), fatty acid synthase (*FAS*), and sterol regulatory element-binding transcription factor 1c (*SREBP1C*). Genes associated with fatty-acid oxidation and mitochondrial fatty-acid transport included carnitine palmitoyltransferase 1A (*CPT1A*) and long-chain acyl-CoA dehydrogenase (*LCAD*). Triglyceride synthesis and lipogenic regulation were assessed using peroxisome proliferator-activated receptor gamma (*PPARG*). Cholinergic markers included choline O-acetyltransferase (*CHAT*), acetylcholinesterase (*ACHE*), cholinergic receptor nicotinic alpha 7 (*CHRNA7*), cholinergic receptor muscarinic M1 (*CHRM1*), cholinergic receptor muscarinic M3 (*CHRM3*), and cholinergic receptor muscarinic M5 (*CHRM5*). Inflammatory markers included tumor necrosis factor (*TNF*), interleukin-1 beta (*IL1B*), and interleukin-6 (*IL6*). GAPDH served as the internal reference gene for normalization, and relative transcript abundance was calculated using the 2^−ΔΔCt^ method (Rao et al., 2013). Primer sequences are provided in Supplementary Table.

### 2.5. Quantification of TNF-α, COX-2, and IL-1β by ELISA

Levels of TNF-α, COX-2, and IL-1β were quantified using human-specific ELISA kits (TNF-α: Invitrogen, Thermo Fisher Scientific, Waltham, MA, USA; COX-2: Sunred Biological Technology Co. Ltd., Shanghai, China; IL-1β: Reed Biotech, Shanghai, China) according to the manufacturers’ instructions. Conditioned media were collected for the quantification of TNF-α and IL-1β by ELISA, whereas COX-2 protein levels were determined in cell lysates. Total protein content was determined using a bicinchoninic acid assay to standardize lysate protein inputs. Each sample was analyzed in triplicate.

### 2.6. Cholinesterase activity assays

Total cholinesterase (ChE) and acetylcholinesterase (AChE) activities in conditioned media were measured using the Ellman method (Ellman et al., 1961). AChE activity was determined in the presence of 10 μM iso-OMPA, a selective inhibitor of butyrylcholinesterase (BChE).

### 2.7. Statistical analysis

Data processing, graph generation, and statistical analyses were performed using GraphPad Prism version 10.0 (GraphPad Software, San Diego, CA, USA). Data were analyzed using one-way ANOVA, followed by Dunnett’s post hoc test. Unless otherwise specified, experiments were conducted in at least three independent biological replicates. The specific statistical tests, sample sizes, and exact p values are reported in the corresponding figure legends.

### 2.8. Molecular modeling of α-LA interactions

Ligand structures were generated and energy-minimized using LigPrep (2025-1; Schrödinger LLC, New York, NY, USA) and MacroModel (2025-1; Schrödinger LLC, New York, NY, USA) with the OPLS4 force field (Lu et al., 2021). The crystal structures of the human α7 nicotinic acetylcholine receptor (α7 nAChR; PDB ID: 8CI2) (Prevost et al., 2023) and rat M3 muscarinic acetylcholine receptor (M3 mAChR; PDB ID: 5ZHP) (Liu et al., 2018) were retrieved from the RCSB protein data bank^1^ (Berman et al., 2000). Protein preparation was performed using the protein preparation wizard implemented in Maestro (2025-1; Schrödinger LLC, New York, NY, USA) in interactive mode (Sastry et al., 2013). During preparation, redundant molecules were removed, hydrogen atoms were added, bond orders and partial charges were assigned, hydrogen-bond networks were optimized, and restrained force-field energy minimization was performed. Receptor grid boxes were generated in Maestro as cubic grids with a volume of 27,000 Å3 and centered at coordinates (107.75, 143.12, 124.63) for α7 nAChR and (−21.15, −48.13, 196.55) for M3 mAChR. Ligands were docked into the receptor binding sites using Glide (2025-1; Schrödinger LLC, New York, NY, USA) in standard precision mode with 100 docking runs per ligand. Binding free-energy (ΔG) values were calculated for the top-ranked ligand–receptor complexes using the MM-GBSA module implemented in Maestro (2025-1; Schrödinger LLC, New York, NY, USA). Prior to α-LA docking, the cocrystallized ligand associated with each receptor structure was redocked, and the resulting poses were compared with their corresponding experimental conformations using root-mean-square deviation (RMSD) analysis. RMSD values of 0.54 Å for α7 nAChR and 0.31 Å for M3 mAChR were obtained, indicating good predictive reliability of the docking protocol.

## 3. Results

### 3.1. Inflammatory profile

**Figure 1:**
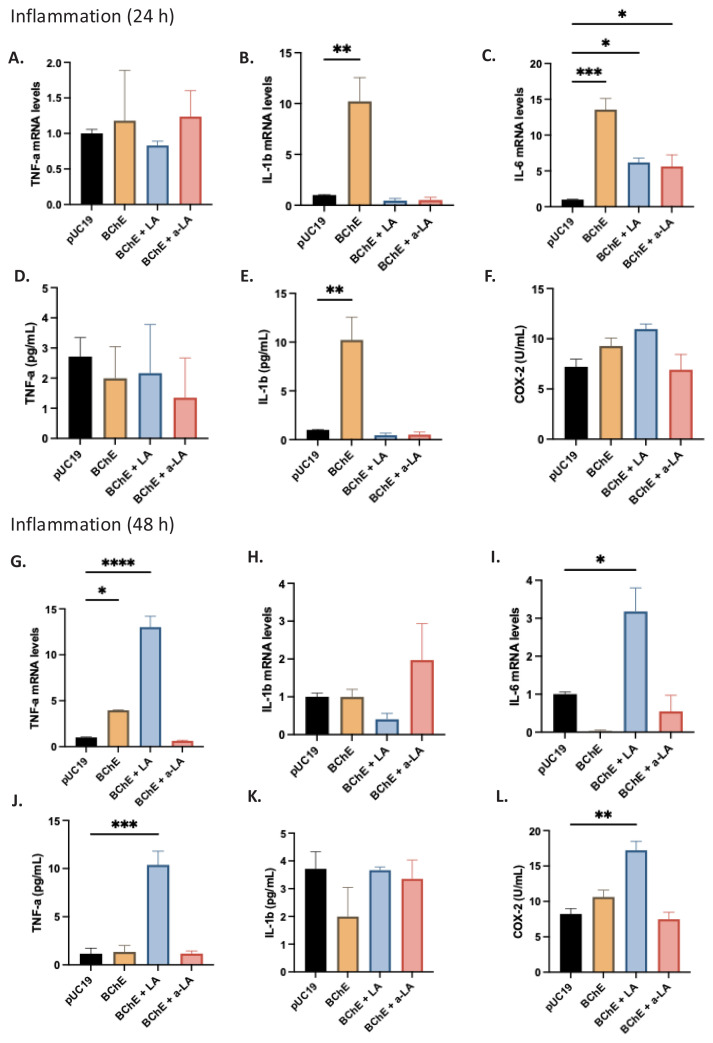
Inflammatory profile in HepG2 cells at 24 and 48 h. At 24 h (A–F), TNF mRNA (A) TNF-α protein levels (D) did not differ significantly among groups. IL-1β mRNA expression (B) was increased in the BChE group, whereas IL6 mRNA expression (C) was elevated following BChE overexpression and was attenuated by cotreatment with LA or α-LA relative to BChE alone. IL-1β protein levels (E) were increased in the BChE group, whereas COX-2 protein levels (F) did not differ significantly among groups. At 48 h (G–L), TNF mRNA expression (G) was increased in the BChE + LA group, whereas IL1β mRNA expression (H) did not differ significantly among groups. IL6 mRNA expression (I) was highest in the BChE + LA group. The transcriptional findings were partially reflected at the protein level. TNF-α protein levels (J) were increased in the BChE + LA group, whereas IL-1β protein levels (K) did not differ significantly among groups. COX-2 protein levels (L) were lower in the BChE + α-LA group than in the BChE + LA group. Groups: pUC19 (control), BChE, BChE + LA, and BChE + α-LA. Data are presented as mean ± SEM (n = 3 independent biological replicates). *, **, ***, and **** indicate p < 0.05, p < 0.01, p < 0.001, and p < 0.0001, respectively.

At 24 h, *TNF* (p = 0.848; Figure 1A), *IL1β* (p = 0.002; Figure 1B), and *IL6* (p = 0.001; Figure 1C) mRNA expression levels were assessed. Whereas TNF transcript levels remained unchanged relative to the pUC19 control group, BChE overexpression increased *IL1β* and *IL6* transcript levels, and these effects were attenuated by cotreatment with either LA or α-LA. At the protein level, TNF-α concentrations remained unchanged at 24 h (p = 0.852; Figure 1D), whereas IL-1β secretion was increased in BChE-overexpressing cells relative to the control group (Figure 1E); this effect was attenuated by cotreatment with either LA or α-LA. COX-2 protein levels did not differ significantly among the groups (p = 0.073; Figure 1F). By 48 h, *TNF* (p < 0.0001; Figure 1G) and *IL1β* (p = 0.31; Figure 1H) mRNA expression levels were evaluated. *TNF* transcript levels were markedly increased by LA cotreatment, whereas IL1β transcript levels remained unchanged across the experimental groups. *IL6* mRNA expression was also increased by LA treatment (p = 0.004; Figure 1I), whereas α-LA attenuated this response. At the protein level, TNF-α concentrations were significantly increased following LA cotreatment (p = 0.012; Figure 1J), whereas IL-1β protein levels did not differ significantly among the groups (p = 0.31; Figure 1K). Finally, COX-2 protein levels were highest in the LA cotreatment group (p = 0.004; Figure 1L), whereas α-LA cotreatment consistently reduced inflammatory marker levels relative to the BChE + LA group.

### 3.2. De novo fatty-acid synthesis

**Figure 2:**
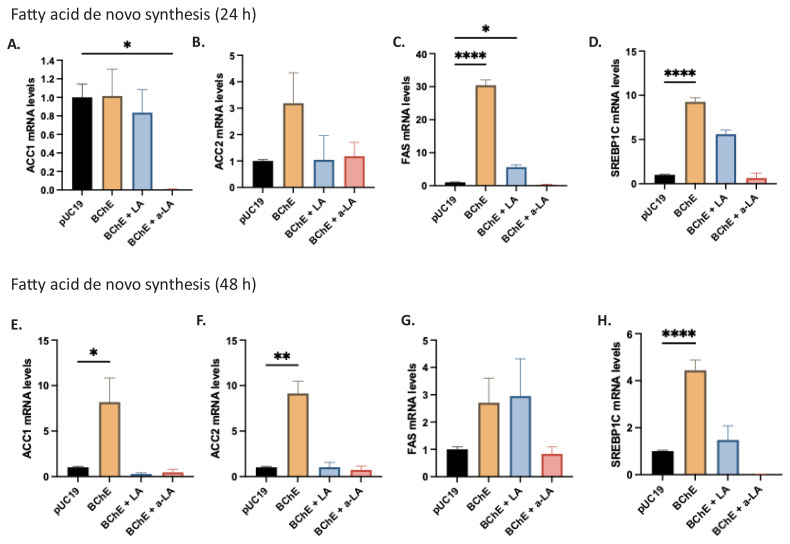
Expression of genes involved in de novo fatty-acid synthesis at 24 h and 48 h. Relative mRNA expression levels of genes involved in de novo fatty-acid synthesis were measured by qPCR after 24 h (A–D) and 48 h (E–H) of treatment. Genes analyzed included *ACC1* (A,E), *ACC2* (B,F), *FAS* (C,G), and *SREBP1C* (D,H). Cells were transfected with pUC19 (control) or BCHE and were treated with linoleic acid (LA) or α-linolenic acid (α-LA), as indicated. At 24 h, BCHE overexpression was associated with increased *ACC1*, *FAS*, and *SREBP1C* mRNA expression relative to the control group, and these increases were attenuated by cotreatment with LA or α-LA. At 48 h, BCHE overexpression was associated with increased *ACC1*, *ACC2*, and *SREBP1C* expression, whereas cotreatment with LA or α-LA attenuated these effects. Data are presented as mean ± SEM (n = 3 independent biological replicates). *, **, ***, and **** indicate p < 0.05, p < 0.01, p < 0.001, and p < 0.0001, respectively.

At 24 h, *ACC1* expression was modestly reduced in the LA cotreatment group (p = 0.036; Figure 2A). At 24 h, ACC2 expression tended to increase following BChE overexpression, although this effect did not reach statistical significance (p = 0.187; Figure 2B). In contrast, *FAS* and *SREBP1C* expression levels were markedly increased (p < 0.0001; Figures 2C and 2D), with *SREBP1C* serving as a master regulator of de novo lipogenesis. Cotreatment with either fatty acid attenuated this BChE-associated lipogenic response, with α-LA producing the most pronounced effect by reducing *FAS* and *SREBP1C* expression to near-baseline levels, whereas LA produced only a partial reduction. By 48 h, *ACC1* (p = 0.036; Figure 2E) and *ACC2* (p = 0.003; Figure 2F) expression levels were significantly increased in the BChE group. These increases were attenuated by LA and were completely abolished by α-LA. At this later time point, *FAS* expression did not differ significantly among the experimental groups (p = 0.286; Figure 2G). In contrast, *SREBP1C* expression remained highest in the BChE group (p < 0.0001; Figure 2H), was partially reduced by LA, and was markedly suppressed by α-LA.

### 3.3. Fatty-acid oxidation–related and triglyceride metabolism–related gene expression

**Figure 3:**
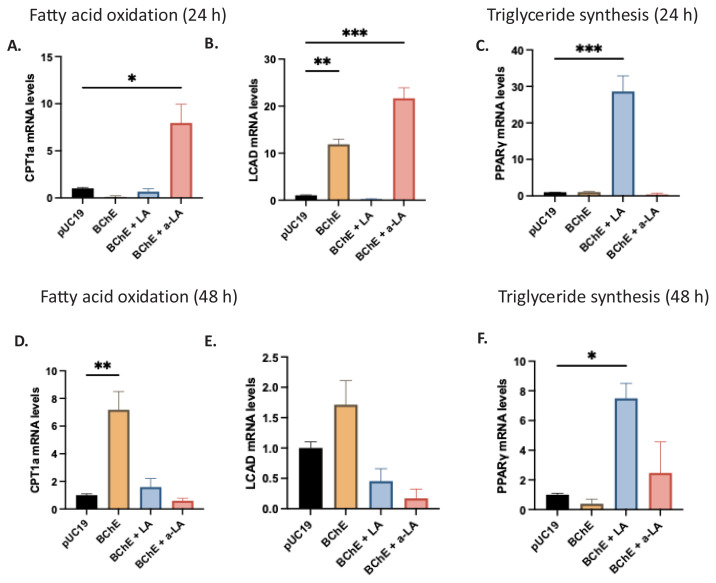
Expression of fatty-acid oxidation–related and triglyceride metabolism–related genes at 24 h and 48 h. At 24 h, *CPT1A* expression was increased in the BChE + LA group (A). *LCAD* expression was increased following BChE overexpression and was further elevated in the BChE + α-LA group (B). *PPARG* expression was markedly increased in the BChE + LA group (C). At 48 h, *CPT1A* expression was increased in the BChE group (D). No significant differences were observed in *LCAD* expression (E). *PPARG* expression remained elevated in the BChE + LA group (F). Data are presented as mean ± SEM (n = 3 independent biological replicates). *, **, ***, and **** indicate p < 0.05, p < 0.01, p < 0.001, and p < 0.0001, respectively.

At 24 h, LA cotreatment was associated with the greatest induction of fatty-acid oxidation–related genes. *CPT1A* (p = 0.014) and *LCAD* (p = 0.0008) expression levels were markedly increased relative to the pUC19 and BChE groups (Figures 3A and 3B), whereas α-LA maintained both transcripts at near-baseline levels. In parallel, *PPARG* expression was markedly increased in the BChE + LA group and remained comparatively low in the other experimental groups (p = 0.0003; Figure 3C). By 48 h, a distinct expression pattern emerged. At 48 h, *CPT1A* expression was highest in the BChE group (p = 0.009; Figure 3D), and both fatty-acid treatments, particularly α-LA, attenuated this response. Differences in LCAD expression were modest and did not reach statistical significance (Figure 3E). *PPARG* expression remained elevated in the BChE + LA group relative to the other experimental conditions, whereas α-LA treatment was associated with only a modest increase (p = 0.0395; Figure 3F).

### 3.4. Cholinergic-associated gene expression

**Figure 4:**
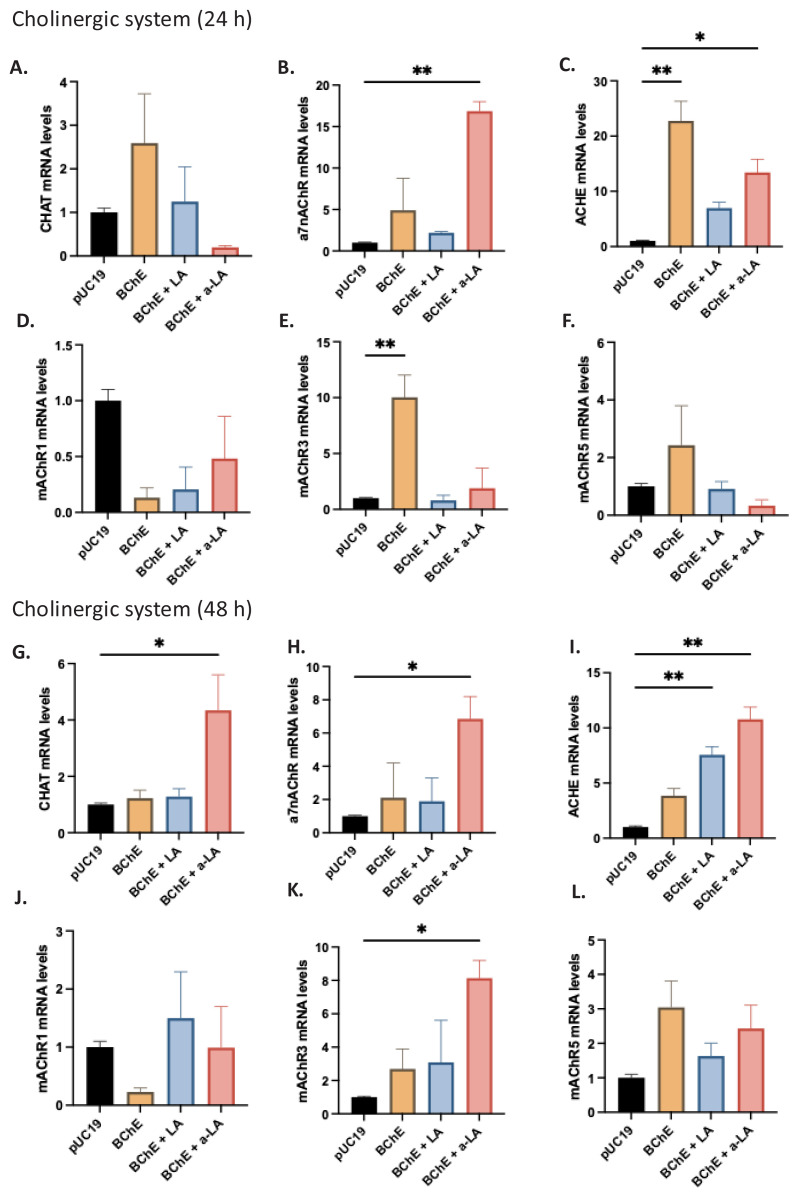
Cholinergic gene expression at 24 and 48 h. At 24 h, *CHAT* expression did not differ significantly among groups (A). *CHRNA7* expression was increased in the BChE + α-LA group (B). *ACHE* expression was increased in the BChE group (C), whereas *CHRM1* expression did not differ significantly among groups (D). *CHRM3* expression was increased in the BChE group (E). *CHRM5* expression did not differ significantly among groups (F). At 48 h, *CHAT* expression was increased in the BChE + α-LA group (G). *CHRNA7* expression was also increased in the BChE + α-LA group (H). ACHE expression was increased in the BChE and BChE + LA groups relative to the control group (I). *CHRM1* expression did not differ significantly among groups (J). *CHRM3* expression was increased in the BChE + α-LA group (K). *CHRM5* expression did not differ significantly among groups (L). Data are presented as mean ± SEM (n = 3 independent biological replicates). *, **, ***, and **** indicate p < 0.05, p < 0.01, p < 0.001, and p < 0.0001, respectively.

At 24 h, *CHAT* expression did not differ significantly among the experimental groups (p = 0.248; Figure 4A). *CHRNA7* expression was markedly increased in the BChE + α-LA group (p = 0.004; Figure 4B), whereas *ACHE* expression was highest in the BChE group and was reduced by both fatty-acid treatments (p = 0.008; Figure 4C). Among the muscarinic receptor genes examined, *CHRM1* expression tended to be lower in the fatty-acid treatment groups than in the pUC19 control group, although this difference did not reach statistical significance (p = 0.152; Figure 4D). *CHRM3* expression was significantly increased following BChE overexpression (p = 0.007; Figure 4E), whereas *CHRM5* expression exhibited a nonsignificant upward trend (p = 0.323; Figure 4F). By 48 h, *CHAT* expression was highest in the BChE + α-LA group (p = 0.026; Figure 4G), accompanied by increased CHRNA7 expression in the same group (p = 0.0396; Figure 4H). In contrast, *ACHE* expression remained elevated in both the BChE + LA and BChE + α-LA groups (p = 0.002; Figure 4I). For the muscarinic receptor genes, *CHRM1* expression was highest in the BChE + LA group; however, this difference was not statistically significant (p = 0.850; Figure 4J). *CHRM3* expression was increased in the BChE + α-LA group (p = 0.022; Figure 4K), whereas differences in *CHRM5* expression did not reach statistical significance across the experimental groups (p = 0.181; Figure 4L).

### 3.5. Cholinesterase activity

**Figure 5:**
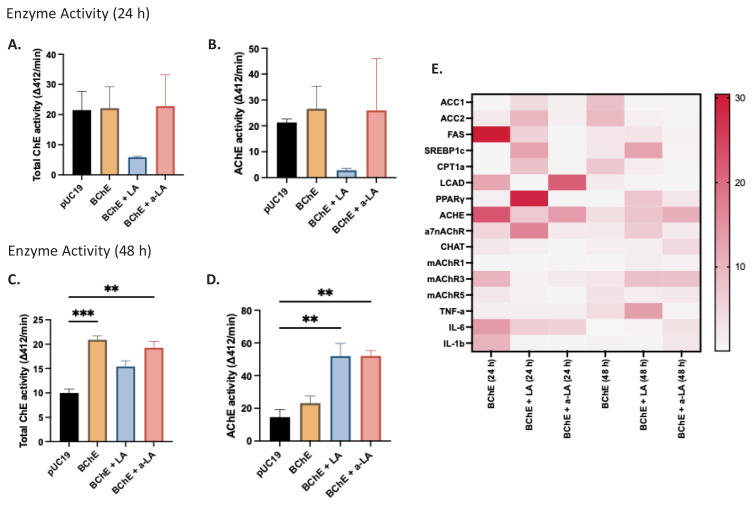
Cholinesterase activity and integrated transcriptional summary at 24 h and 48 h. (A–D) Total ChE and AChE activities were measured in HepG2 cells from four experimental groups: pUC19 (control), BChE, BChE + LA, and BChE + α-LA. Activities were measured using the Ellman assay and are presented as ΔA_412_/min. AChE activity was determined in the presence of iso-OMPA to inhibit BChE activity. At 24 h (A,B), no significant differences were observed in either total ChE activity or AChE activity. At 48 h (C,D), total ChE activity was increased in the BChE and BChE + α-LA groups, whereas AChE activity was increased in the BChE + LA and BChE + α-LA groups relative to the control group. (E) Heatmap summarizing fold changes in the expression of key lipid metabolism–related and cholinergic-associated genes across the indicated experimental conditions and time points, normalized to the corresponding time-matched control group (darker red indicates higher expression). Data are presented as mean ± SEM (n = 3 independent biological replicates). *, **, ***, and **** indicate p < 0.05, p < 0.01, p < 0.001, and p < 0.0001, respectively.

At 24 h, total ChE activity did not differ significantly among the experimental groups (p = 0.508; Figure 5A). At the same time point, specific AChE activity was not significantly altered (p = 0.467; Figure 5B). By 48 h, total ChE activity was significantly increased in the BChE and α-LA treatment groups relative to the control group (p = 0.001; Figure 5C). Similarly, specific AChE activity was significantly higher in the LA- and α-LA-treated groups than in the control groups (p = 0.006; Figure 5D).

### 3.6. Molecular docking

**Figure 6:**
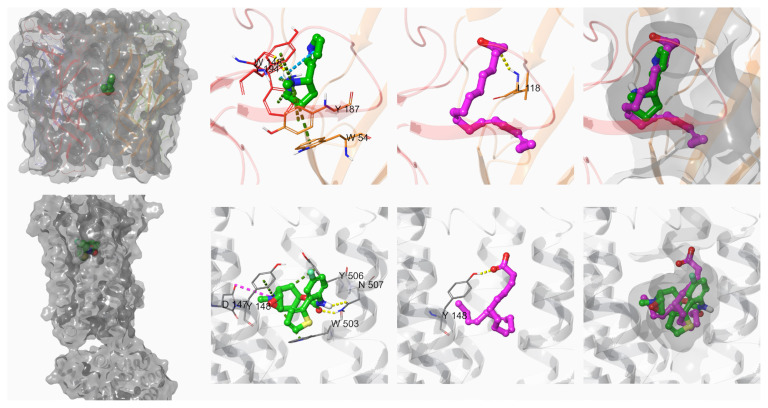
(A) Extracellular domain of the α7 nAChR (backbone shown as colored cartoon representations according to chain identity) with nicotine (green CPK representation) bound in the orthosteric site. (B) Binding mode and electrostatic interactions of nicotine (green stick-and-ball representation) within the α7 nAChR orthosteric site in the cryo-EM structure. (C) Predicted binding mode and electrostatic interactions of α-LA (orange stick-and-ball representation) within the α7 nAChR orthosteric site. (D) Superposition of the experimentally observed nicotine binding pose and the predicted α-LA binding pose within the α7 nAChR orthosteric site. (E) M3 mAChR (backbone shown as a white cartoon representation) with a selective antagonist (green CPK representation) bound in the orthosteric site. (F) Binding mode and electrostatic interactions of the cocrystallized antagonist (green stick-and-ball representation) within the M3 mAChR orthosteric site. (G) Predicted binding mode and electrostatic interactions of α-LA (orange stick-and-ball representation) within the M3 mAChR orthosteric site. (H) Superposition of the experimentally observed antagonist binding pose and the predicted α-LA binding pose within the M3 mAChR orthosteric site.

The homopentameric α7 nAChR is a ligand-gated cation channel that is activated by the binding of ACh to an orthosteric site located at the extracellular interface between two adjacent subunits (Figure 6A). Key residues within the orthosteric site include W148, Y187, and Y194 of the principal subunit and W54 of the complementary subunit; these residues have been reported to interact with nicotine, a prototypical agonist of the nicotinic receptor family (Figure 6B). Occupancy of the orthosteric binding pocket by α-LA was predicted, with its carboxylate group oriented toward L118 of the complementary subunit and stabilized by a putative hydrogen bond (Figure 6C). Notably, L118 has also been reported to participate in interactions with the pyridine ring of nicotine. The alkenyl chain adopted a bent conformation around C9–C12, allowing partial overlap with the bound conformation of the cocrystallized antagonist, particularly along the C3–C12 region (Figure 6D).

The orthosteric binding site of the M3 mAChR, located within the transmembrane (TM) domain (Figure 6E), contains Asp147, a key residue involved in the recognition of agonists and antagonists through ionic interactions with quaternary ammonium groups. Additional residues, including Tyr148, Tyr506, Tyr529, and Ser151, participate in ligand recognition through electrostatic interactions, as illustrated for the cocrystallized antagonist in Figure 6F. Binding of α-LA within the orthosteric pocket was predicted, with its carboxylate group positioned toward the extracellular side of the TM domain and stabilized by a putative hydrogen bond with Tyr148 (Figure 6G). The fatty-acid chain adopted a spiral conformation between C5 and C13, occupying a volume comparable to that of the cocrystallized antagonist and enabling van der Waals contacts with residues lining the binding pocket (Figure 6H; Supplementary Figure). The calculated ΔG values for the predicted α-LA poses were less favorable than those of the cocrystallized reference ligands, suggesting moderate-to-low binding affinity for both receptors (Table).

## 4. Discussion

Essential fatty acids, LA (18:2 n-6) and α-LA (18:3 n-3), must be obtained from the diet and serve as the principal precursors of omega-6 and omega-3 PUFAs, respectively. Following dietary intake, PUFAs are incorporated into cellular membranes, where they contribute not only to membrane fluidity but also to the regulation of diverse biological processes, including cytokine release, cell motility, cardiac excitability, and platelet aggregation. In the present HepG2 model, LA and α-LA produced distinct, time-dependent effects on hepatocellular lipid metabolism and inflammatory status, with cholinergic-associated pathways potentially contributing to these outcomes (Djuricic and Calder, 2021).

Hepatic production of BChE is the primary source of circulating enzyme. In clinical practice, plasma BChE activity is primarily measured as an indicator of hepatic synthetic capacity (Massoulié et al., 1993). Reduced BChE levels occur in liver failure, whereas the clinical significance of elevated levels remains unclear. Heni et al. (2025) recently reported a positive correlation between hepatic triglyceride content and *BCHE* mRNA expression, the transcript encoded by the gene responsible for circulating BChE production. Evidence from dos Santos et al. (2017) demonstrated that dietary energy restriction decreases BChE activity and that the CHE2 C5+ phenotype attenuates this effect, highlighting a genetic contribution to the relationship between ChE activity and metabolic regulation. Similarly, in the present study, BChE plasmid transfection was associated with increased expression of genes involved in fatty-acid synthesis and triglyceride metabolism. Alterations in cholinesterase activity and cholinergic-associated gene expression were associated with changes in hepatocellular metabolic plasticity, potentially influencing the balance between energy storage and utilization. This observation is consistent with the hepatic origin of BChE, its reported associations with systemic metabolic traits, and its role in ghrelin metabolism, supporting the concept of an integrated neurometabolic axis linking appetite-related and hormonal signals to hepatocellular lipid regulation (Yao et al., 2016). Within this framework, cholinergic-associated mechanisms may influence not only lipid metabolism but also inflammatory responses, potentially complementing PUFA-derived eicosanoid and oxylipin pathways that contribute to progression toward, or protection from, steatohepatitis.

The early α-LA-associated phenotype (↓lipogenesis and expression changes consistent with enhanced fatty-acid oxidation) is compatible with the induction of n-3-responsive regulators that favor fatty-acid catabolism and the generation of antiinflammatory lipid mediators, whereas the attenuation observed at later time points may reflect feedback mechanisms or substrate depletion that limit sustained responses. Conversely, the delayed proinflammatory transcriptional response observed following LA treatment is consistent with the accumulation of n-6-derived lipid mediators that may amplify inflammatory signaling over time. Changes in cholinesterase activity and cholinergic-associated gene expression may be relevant to mechanisms linked to cholinergic antiinflammatory signaling (Pavlov et al., 2003) and/or acetylcholine receptor–associated pathways. Within this framework, cholinergic-associated mechanisms may contribute to the favorable α-LA-associated response and attenuate the delayed proinflammatory response associated with LA.

In the context of MASLD, these findings suggest that (i) the n-6/n-3 balance is important, (ii) exposure duration influences cellular responses, and (iii) cholinergic-associated factors may contribute to the observed metabolic and inflammatory phenotypes. From a translational perspective, two complementary approaches emerge: (1) interventions targeting lipid composition and (2) strategies aimed at modulating cholinergic-associated pathways. Because α-LA and LA exhibited divergent, time-dependent effects in the present model, both dosing and exposure duration should be considered explicitly in future translational studies rather than being treated as static variables.

Dietary choline is the principal precursor for hepatic phosphatidylcholine (PC) via the CDP-choline pathway and, together with the phosphatidylethanolamine N-methyltransferase (PEMT) pathway, sustains membrane integrity and very-low-density lipoprotein (VLDL) secretion (Noga and Vance, 2003). Insufficient choline intake reduces hepatic PC availability, predisposing to steatosis and endoplasmic reticulum stress. Notably, polyunsaturated PC pools are reduced in MASLD, particularly in MASH, a pattern that has been associated with impaired hepatocellular function and altered membrane dynamics (Ooi et al., 2021). Consistency with this framework was observed in two respects. First, α-LA was associated with early reductions in *SREBP1C* and *ACC1* expression, together with expression changes consistent with enhanced fatty-acid oxidation. These changes may reduce lipotoxic stress on cellular membranes under conditions of limited PC availability. In contrast, LA exhibited a delayed proinflammatory profile that may exacerbate membrane stress when polyunsaturated PC levels are reduced. Second, time-dependent changes in cholinergic-associated markers were observed. *CHAT* and *CHRNA7* expression were increased, most prominently in the BChE + α-LA group, together with increased ACHE expression and AChE activity at 48 h. This pattern may reflect coordinated regulation of cholinergic-associated pathways, whereby α-LA is associated with increased expression of genes involved in ACh synthesis and α7 receptor signaling, together with increased hydrolytic capacity. One possible interpretation is that increased ACh turnover may facilitate choline recycling, thereby contributing to the maintenance of intracellular choline availability for the CDP-choline pathway and PC synthesis. Choline, ACh, and PC flux were not directly quantified; thus, targeted studies will be necessary to determine whether this mechanism contributes to the observed phenotype. Finally, because PC composition and cholinergic signaling are metabolically interconnected, reduced levels of polyunsaturated PC (as observed in MASH) may increase membrane vulnerability and influence receptor function. In parallel, limited choline availability could constrain both PC remodeling and ACh synthesis, potentially attenuating α7 receptor–associated antiinflammatory responses despite increased *CHRNA7* expression.

It may also be hypothesized that α-LA directly interacts with cholinergic receptors as a lipidic allosteric modulator. Multiple allosteric pockets capable of accommodating lipophilic ligands have been identified in α7 nAChR structures (Burke et al., 2024). In addition, nAChR function has been shown to be sensitive to the surrounding polyunsaturated lipid environment, rendering both direct receptor binding and membrane-mediated mechanisms plausible (Sharp et al., 2019). Similarly, well characterized allosteric sites have been described for the M3 muscarinic acetylcholine receptor (M3 mAChR). Furthermore, incorporation of unsaturated fatty acids into cellular membranes has been reported to alter muscarinic agonist affinity, and structural as well as computational studies have supported the presence of dynamic, lipid-exposed allosteric pockets in mAChRs (Stahl et al., 2011). However, it should be noted that these considerations are based on structural and theoretical evidence, and do not constitute direct proof of receptor binding or functional engagement in the present cellular system. Accordingly, these observations should be regarded as complementary and hypothesis-generating and will require further experimental validation.

Previous studies have demonstrated that human BChE participates in lipid handling through lipid-hydrolase activity and that essential fatty acids such as LA and α-LA modulate BChE expression in hepatic cells, thereby linking the enzyme to metabolic signaling (Gok et al., 2024; Gok et al., 2023; Gok et al., 2025). Previous findings have indicated that both LA and α-LA inhibit BChE and AChE activity, with LA exhibiting the stronger inhibitory effect (Akay et al., 2023; Gok et al., 2025). Consistent with a homeostatic response to reduced ChE activity, ACHE mRNA expression was increased in both the BCHE + LA and BCHE + α-LA groups, with the greater increase being observed in the BCHE + α-LA group. This observation suggests that transcriptional upregulation of ACHE is not solely proportional to catalytic inhibition and may involve additional regulatory mechanisms. Potential contributing mechanisms include PUFA-mediated signaling through lipid-sensing receptors that may regulate cholinergic gene expression; alterations in membrane composition that may influence cholinergic receptor activity and feedback regulation of ACHE transcription; differences in cytokine profiles, with α-LA being associated with greater suppression of TNF-α, IL-6, and IL-1β; and posttranscriptional effects on ACHE mRNA stability. Collectively, these findings suggest that enzyme inhibition may initiate a compensatory increase in ACHE expression, whereas α-LA may additionally influence regulatory pathways that enhance this response beyond that expected from enzyme inhibition alone.

In the present study, BCHE overexpression was employed to modulate ChE levels in HepG2 cells, and the resulting increase in enzymatic activity supported the functional relevance of this approach. This relationship between gene expression and enzymatic activity suggests that the overexpression strategy was effectively translated into measurable biochemical changes, although additional factors, including posttranslational modifications and enzyme stability, may also have contributed. A transfection control, pUC19, was used to account for nonspecific effects associated with plasmid introduction. Although this approach allows normalization of transfection-related responses, the use of an empty mammalian expression vector would constitute a more conventional control and should be considered in future studies.

The interpretation that ChE activity may reflect alterations in lipid handling is supported by a randomized, double-blind study conducted in sedentary young men. In that study, 4 weeks of endurance exercise, with or without conjugated linoleic acid (CLA) supplementation, reduced plasma BChE activity as well as insulin and leptin levels, increased postheparin lipoprotein lipase (LPL) activity, and, in the CLA-supplemented group, decreased LDL, VLDL, and triglyceride concentrations (Bulut et al., 2013). Although CLA differs structurally and metabolically from α-LA, and the cited intervention included an exercise component, these findings support a broader association between unsaturated fatty acid exposure, lipoprotein metabolism, and ChE activity. This interpretation is consistent with the α-LA-associated changes observed in cholinergic-related markers in the present study.

A major strength of this study is the side-by-side evaluation of n-3 and n-6 PUFAs under both acute and prolonged exposure conditions, combined with integrated assessments of lipid synthesis, fatty-acid oxidation–related markers, triglyceride metabolism, inflammatory markers, and cholinergic-associated parameters within a single hepatocyte model. However, several limitations should be acknowledged. The use of a transformed HepG2 cell line does not recapitulate the multicellular, metabolic, inflammatory, and fibrotic complexity of MASLD/MASH observed in vivo. Although widely used as a model of hepatic metabolism, this system cannot fully recapitulate normal hepatocyte physiology. Therefore, the findings should be interpreted as evidence of potential mechanistic involvement rather than direct disease modeling and should be validated in primary hepatocytes and in vivo models.

In addition, the selection of only 24 h and 48 h time points limited the ability to fully resolve temporal dynamics, and the absence of dose–response analyses restricted the assessment of concentration-dependent effects. Furthermore, the PUFA panel was limited to LA and α-LA; inclusion of additional fatty acids such as arachidonic acid would provide a more comprehensive view of lipid-mediated regulation. Moreover, comprehensive protein-level validation of key targets, including BChE and cholinergic-associated markers, was not performed. In addition, direct functional assessments of lipid metabolism were not conducted. These limitations reduce the ability to confirm whether the observed transcriptional changes translated into corresponding functional effects.

From 24 to 48 h, α-LA cotreatment in BChE-overexpressing cells shifted the transcriptional profile toward a pattern consistent with reduced lipogenesis and enhanced fatty-acid oxidation–related gene expression, as reflected by increased CPT1A and LCAD expression relative to ACC1, ACC2, FAS, and SREBP1C. This transcriptional shift was associated with increased cholinergic-associated markers, including AChE activity, *CHAT* expression, and *CHRNA7* expression, together with reduced expression of proinflammatory mediators, including *TNF*, *IL6*, and *IL1β*. These findings suggest a coordinated relationship between cholinergic-associated changes and transcriptional responses related to metabolic and inflammatory pathways. In contrast, LA exhibited a more transient early response, whereas α-LA-associated effects appeared to be more sustained, highlighting both time-dependent and PUFA-specific differences.

Future studies should incorporate broader time-course and dose–response designs, targeted lipidomic analyses, and mechanistic investigation of specific cholinergic-associated pathways. Extension to coculture systems and in vivo models will be important to validate these interactions in more physiologically relevant settings.

In summary, PUFA class and exposure duration jointly influenced hepatic metabolic and inflammatory responses, with cholinergic-associated mechanisms representing a potential modulatory component. These cholinergic–lipid interactions may represent translationally relevant targets in MASLD, emphasizing the potential importance of both fatty-acid composition and exposure duration in the development of therapeutic strategies.

## Supplementary materials

**Supplementary Table:** List of qPCR primers

human *GAPDH* F1	5′CATGTACGTTGCTATCCAGGC3′
human *GAPDH* R1	5′CTCCTTAATGTCACGCACGAT3′
human *TNF*-F1	5′ATGAGCACTGAAAGCATGATC3′
human *TNF*-R1	5′GAGGGCTGATTAGAGAGAGGT3′
human *IL-6*-F1	5′ACTCACCTCTTCAGAACGAATTG3′
human *IL-6*-R1	5′CCATCTTTGGAAGGTTCAGGTTG3′
human *IL-1b*-F1	5′ CCACAGACCTTCCAGGAGAATG3′
human *IL-1b*-R1	5′ GTGCAGTTCAGTGATCGTACAGG3′
human *CHAT-F1*	5′ CCAGCCCCATTCTTTCCACT3′
human *CHAT-R1*	5′ CCAGCTAGTGGGCTTGTTGT3′
human *CHRM1-F1*	5′ACCCTACAGACCCCTCTTCAG3′
human *CHRM1-R1*	5′ GAGTCACGGAGAAGTAGCGG3′
human *CHRM3-F1*	5′ CTGTCTGCCCTAGACTCCAC3′
human *CHRM3-R1*	5′ CATCGGAGGGGCTGTGTATC3′
human *CHRM5-F1*	5′ CCACCATCACTTTTGGCACTGC3′
human *CHRM5-R1*	5′ AGGTCCTTGGTTCGCTTCTCTG3′
human *CHRN7-F1*	5′ GACTCAACATGCGCTGCTC3′
human *CHRN7-R1*	5′ TATGCCTGGAGGCAGGTACT3′
human *ACHE-F1*	5′ AATGGGAGTGGAAAGCAGGAT3′
human *ACHE-R1*	5′ GTGAGGGAGAGCAAGAGTCAA3′
human *ACC1-F1*	5′ GAGGGCTAGGTCTTTCTGGAAG3′
human *ACC1-R1*	5′ CCACAGTGAAATCTCGTTGAGA3′
human *ACC2-F1*	5′ GCCAGAAGCCCCCAAGAAAC3′
human *ACC2-R1*	5′ CGACATGCTCGGCCTCATAG3′
human *FAS-F1*	5′AGCTGCCAGAGTCGGAGAAC3′
human *FAS-R1*	5′ TGTAGCCCACGAGTGTCTCG3′
human *SREBP1c-F1*	5′ GCGGAGCCATGGATTGCAC3′
human *SREBP1c-R1*	5′ CTCTTCCTTGATACCAGGCCC3′
human *CPT1a-F1*	5′ TGAGCGACTGGTGGGAGGAG3′
human *CPT1a-R1*	5′ GAGCCAGACCTTGAAGTAGCG3′
human *LCAD-F1*	5′ GGTGTTCATCAGTAATGGGTCAT3′
human *LCAD-R1*	5′ CACTGTCTGTAGGTGAGCAACTG3′
human *PPARG-F1*	5′ GAACAGATCCAGTGGTTGCAG3′
human *PPARG-R1*	5′ GGCATTATGAGACATCCCCAC3′

Supplementary Figure Two-dimensional interactions diagrams for the predicted binding mode of α-LA in the orthosteric site of nAChRa7 (A) and mAChR3 (B).

## Figures and Tables

**Table:** Predicted ΔG values for α-LA–receptor complexes (kcal/mol).

Compound	α7 nAChR	M3 mAChR
α-LA	−28.3	−50.0
Nicotine	−48.7	-
mAChR antagonist	-	−91.5

## Data Availability

The data underlying this study are available from the corresponding author upon reasonable request.

## Footnotes

1 RCSB PDB (2025). RCSB Protein Data Bank [online]. Website: https://www.rcsb.org/ [accessed 29 August 2025].
