# Supplementary figures and images for: An in vitro mechanistic study of cholinergic-associated mechanisms relevant to MASLD in HepG2 cells
Source: Turk J Biol. 2026 Apr 21;50(3):245–58. doi: 10.55730/1300-0152.2806 (PMC13398593; doi:10.55730/1300-0152.2806)

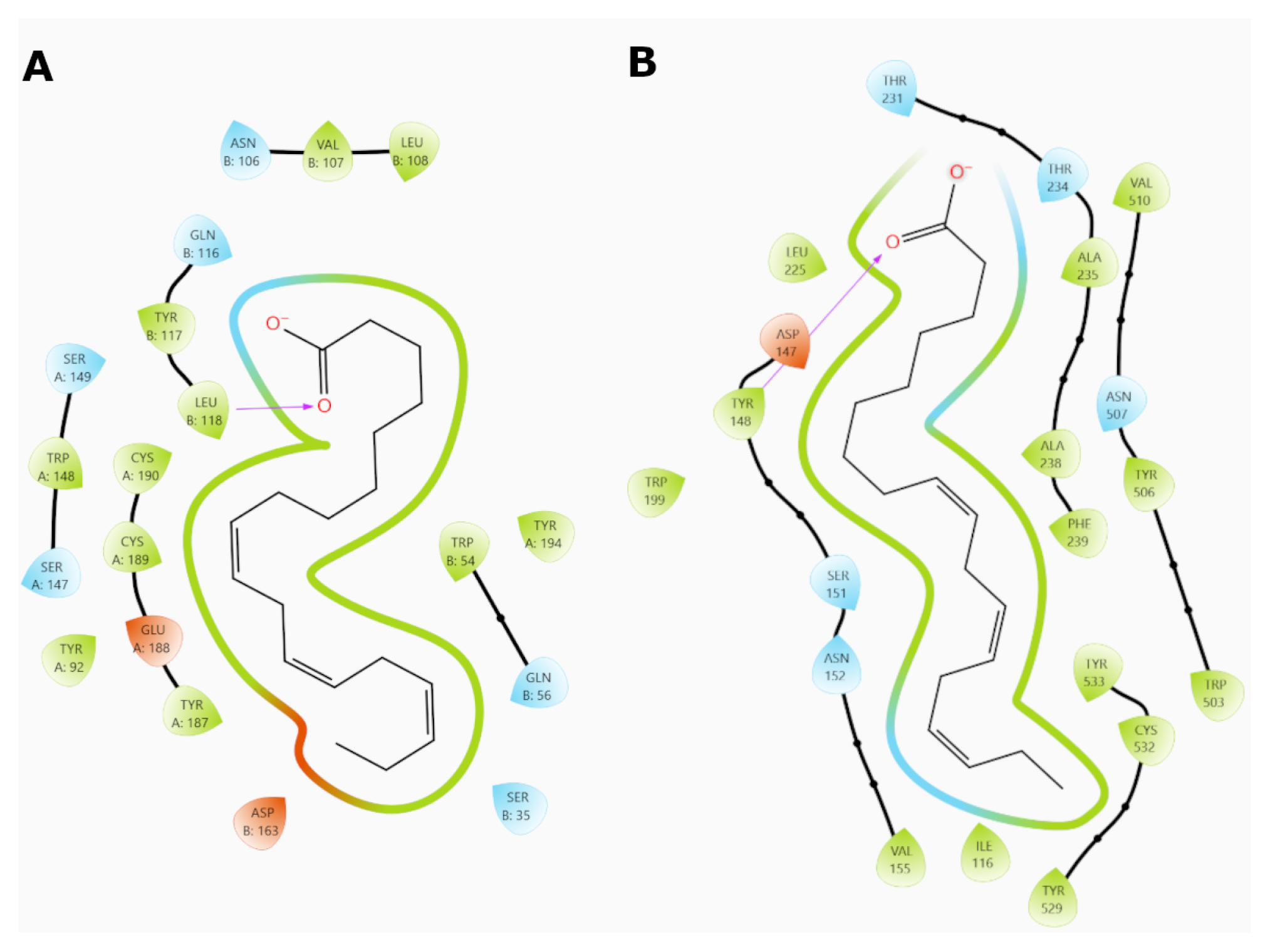

Supplement: Supplementary Figure — Two-dimensional interactions diagrams for the predicted binding mode of α-LA in the orthosteric site of nAChRa7 (A) and mAChR3 (B). [file tjb-50-03-245s1.tif]
